# Determinants of coronavirus disease 2019 infection by artificial intelligence technology: A study of 28 countries

**DOI:** 10.1371/journal.pone.0272546

**Published:** 2022-08-26

**Authors:** Hsiao-Ya Peng, Yen-Kuang Lin, Phung-Anh Nguyen, Jason C. Hsu, Chun-Liang Chou, Chih-Cheng Chang, Chia-Chi Lin, Carlos Lam, Chang-I Chen, Kai-Hsun Wang, Christine Y. Lu

**Affiliations:** 1 International PhD Program in Biotech and Healthcare Management, College of Management, Taipei Medical University, Taipei, Taiwan; 2 Biostatistics Center, Office of Data Science, Taipei Medical University, Taipei, Taiwan; 3 Clinical Data Center, Office of Data Science, Taipei Medical University, Taipei, Taiwan; 4 Research Center of Health Care Industry Data Science, College of Management, Taipei Medical University, Taipei, Taiwan; 5 Clinical Big Data Research Center, Taipei Medical University Hospital, Taipei Medical University, Taipei, Taiwan; 6 Department of Healthcare Information & Management, Ming Chuan University, Taoyuan, Taiwan; 7 Department of Thoracic Medicine, Taipei Medical University Hospital, Taipei Medical University, Taipei, Taiwan; 8 Division of Pulmonary Medicine, Department of Internal Medicine, Shuang Ho Hospital, Taipei Medical University, New Taipei City, Taiwan; 9 Emergency Department, Department of Emergency and Critical Care Medicine, Wan Fang Hospital, Taipei Medical University, Taipei, Taiwan; 10 Department of Emergency, School of Medicine, College of Medicine, Taipei Medical University, Taipei, Taiwan; 11 Graduate Institute of Injury Prevention and Control, College of Public Health, Taipei Medical University, Taipei, Taiwan; 12 Department of Healthcare Administration, School of Management, Taipei Medical University, Taipei, Taiwan; 13 Graduate Institute of Business Administration, College of Management, Fu Jen Catholic University, New Taipei City, Taiwan; 14 Department of Population Medicine, Harvard Medical School and Harvard Pilgrim Health Care Institute, Boston, MA, United States of America; Shahid Beheshti University of Medical Sciences, ISLAMIC REPUBLIC OF IRAN

## Abstract

**Objectives:**

The coronavirus disease 2019 pandemic has affected countries around the world since 2020, and an increasing number of people are being infected. The purpose of this research was to use big data and artificial intelligence technology to find key factors associated with the coronavirus disease 2019 infection. The results can be used as a reference for disease prevention in practice.

**Methods:**

This study obtained data from the "Imperial College London YouGov Covid-19 Behaviour Tracker Open Data Hub", covering a total of 291,780 questionnaire results from 28 countries (April 1~August 31, 2020). Data included basic characteristics, lifestyle habits, disease history, and symptoms of each subject. Four types of machine learning classification models were used, including logistic regression, random forest, support vector machine, and artificial neural network, to build prediction modules. The performance of each module is presented as the area under the receiver operating characteristics curve. Then, this study further processed important factors selected by each module to obtain an overall ranking of determinants.

**Results:**

This study found that the area under the receiver operating characteristics curve of the prediction modules established by the four machine learning methods were all >0.95, and the RF had the highest performance (area under the receiver operating characteristics curve is 0.988). Top ten factors associated with the coronavirus disease 2019 infection were identified in order of importance: whether the family had been tested, having no symptoms, loss of smell, loss of taste, a history of epilepsy, acquired immune deficiency syndrome, cystic fibrosis, sleeping alone, country, and the number of times leaving home in a day.

**Conclusions:**

This study used big data from 28 countries and artificial intelligence methods to determine the predictors of the coronavirus disease 2019 infection. The findings provide important insights for the coronavirus disease 2019 infection prevention strategies.

## Introduction

The coronavirus disease 2019 (COVID-19; also known as severe acute respiratory syndrome coronavirus-2 (SARS-CoV-2)) pandemic has spread rapidly around the world, causing global panic and affecting all aspects of people’s lives and the economy since December 2019. As of July 2021, there have been more than 188 million confirmed COVID-19 cases worldwide and at least 4.06 million deaths [1]. Identifying high-risk groups, taking preventive measures as early as possible, and caring for those who may get sick are important goals for preventing further spread of the global COVID-19 pandemic.

Traditionally, logistic regression in basic statistical methodology has been often used to explore which key influencing factors have a significant correlation with the occurrence of diseases, and thus inform prevention efforts. With the rise of artificial intelligence (AI) in recent years and the development of various algorithms, including AI-based machine learning and deep learning algorithms, researchers can use data obtained to build more accurate prediction models [2]. A prediction module generated using only a single algorithm based on a certain operation logic may not be the most suitable module. Integrating multiple prediction models using multiple algorithms based on various operational logics can generate more comprehensive, complete and objective results.

Researchers are increasingly using AI methods to predict and prevent the occurrence of diseases. Regarding the new global COVID-19 pandemic, medical and academic professionals around the world have also adopted various machine learning and deep learning methods to conduct research on preventing and treating COVID-19. For example, previous study determined weather and climate conditions, such as temperature and humidity, that might affect spread of the COVID-19 virus [3]. AI technology were also applied on medical images (chest x-ray image) to predict whether patients were infected [4], to track the chain of virus transmission, and to assist in the development of vaccines and drugs [5]. Demographic data (ex. age) and clinical data (ex. renal function and the results of COVID-19 RT-PCR tests) were used as predictive indicators to assist in diagnosis [6, 7]. Besides, the combination of modern medical and AI technologies greatly improved the screening, prediction, and tracking of virus contacts, as well as increased the reliability of vaccine and medication development [8, 9]. Many studies also focus on confirmed COVID-19 patients, using machine learning methods to build predictive models for disease prognosis, including severity or mortality [10–12]. Furthermore, some scholars have used AI technologies to predict the development trend of the spread [13, 14] and the health system failure [15] of COVID-19 from the perspective of public health.

None of the abovementioned studies used data from multiple countries and multiple algorithms. To help fill the gap in knowledge, this study investigated the factors associated with COVID-19 infection using big data from multiple countries and multiple algorisms. The current study has two purposes. The first goal was to use machine learning methods to generate a predictive model for COVID-19 infection, and to use simple information to preliminarily check whether an infection is possible. The second objective was to determine important features of COVID-19 infection, and propose precautions and preventive measures to the public based on the results. This study used publicly available questionnaire survey data around the world, which included basic information, living habits, disease history, and symptoms of respondents from 28 countries. The predictive model established by AI technology can help us understand the determinants of COVID-19 infection, and avoid unnecessary hospital visits and nosocomial infections.

## Methods

This section includes data sources, cohort selection, descriptive statistics, algorithms used in this study, methods of comparing results obtained from different algorisms, and the way to find key determinants.

### Data sources

Data used in this study were from the Imperial College London YouGov Covid-19 Behavior Tracker Data Hub. YouGov partnered with the Institute of Global Health Innovation at Imperial College London to gather global insights on people’s behaviors in response to COVID-19. The data in this database came from results of a questionnaire survey of people in 28 countries [16]. Use of data from online open databases for research purposes is exempt from review by the Institutional Review Board (IRB) in Taiwan because the data used is public information.

This study collected data from the above database during April 1, 2020~August 31, 2020. Based on the results of the literature review, we applied a clinical perspective and consulted with clinicians and experts to determined 52 factors (including basic characteristics, lifestyle habits, disease histories, and symptoms) that may lead to COVID-19 infection to build predictive models. Four categories of possible influencing factors were collected. The first category consisted of basic characteristics, including gender, age, number of people in the household, number of children in the household, and country. The second category was lifestyle habits, including number of times washing, sanitizer washing, soap washing, frequency of cleaning, eating alone, sleeping alone, frequency of mask wearing, frequency of covering the nose and mouth, the number of contacts with people inside the home, the number of contacts with people outside the home, number of times of leaving home in a day, avoiding having guests, avoiding contacting people, avoiding going outside, avoiding going to shops, avoiding going to the hospital, avoiding taking public transportation, avoiding small social gatherings, avoiding medium-sized social gatherings, avoiding large-sized social gatherings, avoiding crowded areas, avoiding touching objects, self-isolating, having difficulties isolating, being willing to isolate, and whether the family had been tested. The third category was disease history, including acquired immune deficiency syndrome (AIDS), arthritis, asthma, cancer, cystic fibrosis chronic obstructive pulmonary disease, diabetes, epilepsy, heart disease, hyperlipidemia, hypertension, mental disease, multiple sclerosis, not willing to say, and no disease. The last category was symptoms, including cough, fever, loss of smell, loss of taste, having difficulty breathing, and no symptoms (see S1 Appendix). In total, 52 possible influential factors were assessed in this study.

### Cohort selection

This study retrieved original data of 315,276 interviewees from the above database (during April 1~August 31, 2020). After excluding missing data (*n* = 10,106) and outliers (*n* = 13,390), 291,780 people remain in this study. Outliers include unreasonable data such as washing more than 50 times a day, leaving home more than 20 times a day, etc. This study finally selected cases from 28 countries and used a total of 52 influencing variables to establish a prediction module for COVID-19 infection (see S1 Appendix).

Among the data of the 291,780 cases, only 3,179 were COVID-infected patients (positive samples), and the other 288,601 were non-infected patients (negative samples). Due to the large difference between the two groups of people, the prediction module established by this imbalance might not be accurate. Therefore, this study used the Synthetic Minority Over-sampling Technique (SMOTE) [17] method to generate similar synthetic samples to resolve this data imbalance problem. SMOTE was used to generate additional synthetic positive samples with similar distributions based on the distribution characteristics of the original positive sample. After the samples in this study were processed by SMOTE, the final number of positive samples was 12,716, and the number of negative samples was 14,305. Differences between variables in the two groups are shown in Table 1 (continuous variables) and Table 2 (categorical variables).

**Table 1:** Study group comparison of continuous variables.

Numeric Variables		COVID-19 infection				
		Yes (N = 12,716)		No (N = 14,305)		p-value
		Mean	SD	Mean	SD	
**Basic characteristics**						
	age	32.96	9.41	42.76	16.33	<0.01
**Lifestyle habits**						
	number of times of washing	7.59	5.43	9.75	6.99	<0.01
	sanitizer washing	1.67	0.88	1.94	1.18	<0.01
	soap washing	1.73	0.96	1.45	0.8	<0.01
	frequency of cleaning	1.7	0.88	2.29	1.22	<0.01
	eating alone	1.95	1.05	3.46	1.59	<0.01
	sleeping alone	1.8	1.05	3.54	1.69	<0.01
	frequency of mask wearing	1.51	0.87	2.28	1.67	<0.01
	frequency of covering the nose and mouth	1.67	0.94	1.43	0.88	<0.01
	the number of contacts with people inside the home	4.23	2.01	3.45	2.21	<0.01
	the number of contacts with people outside the home	4.86	4.79	6.85	9.15	<0.01
	number of times of leaving home in a day	3.61	2	2.44	1.63	<0.01
	avoiding having guests	1.91	1.01	2.05	1.3	0.457
	avoiding contacting people	1.72	0.95	1.65	1.2	<0.01
	avoiding going outside	1.84	0.97	2.39	1.29	<0.01
	avoiding going to shops	1.93	0.99	2.52	1.25	<0.01
	avoiding going to the hospital	1.87	1.1	2.09	1.42	0.501
	avoiding taking public transportation	1.72	0.97	1.88	1.33	<0.01
	avoiding small social gatherings	1.89	0.99	2.21	1.34	<0.01
	avoiding medium-sized social gatherings	1.85	1.01	1.91	1.25	<0.01
	avoiding large-sized social gatherings	1.85	0.98	1.63	1.14	<0.01
	avoiding crowded areas	1.7	0.91	1.68	1.03	<0.01
	avoiding touching objects	1.67	0.94	2	1.15	<0.01

**Table 2:** Study group comparison of categorical variables.

Categorical Variables		COVID-19 infection				
		Yes (N = 12,716)		No (N = 14,305)		p-value
		n	%	n	%	
**Basic characteristics**						
	gender(male)	8006	63.00%	7148	50.00%	<0.01
	the number of people in the household					
	0 or not sure	279	2.20%	249	1.70%	<0.01
	1	2355	18.50%	1981	13.80%	<0.01
	2	1485	11.70%	3662	25.60%	<0.01
	3	1675	13.20%	2934	20.50%	<0.01
	4	1843	14.50%	2782	19.40%	<0.01
	5	2121	16.70%	1494	10.40%	<0.01
	6	830	6.50%	651	4.60%	<0.01
	7	640	5.00%	279	2.00%	<0.01
	8 or more	1488	11.70%	273	1.90%	<0.01
	number of children in the household					
	0 or not sure	2505	19.70%	7532	52.70%	<0.01
	1	3929	30.90%	3280	22.90%	<0.01
	2	2771	21.80%	2146	15.00%	<0.01
	3	1011	8.00%	713	5.00%	<0.01
	4	627	4.90%	337	2.40%	<0.01
	5	5	0.00%	2	0.00%	<0.01
	6 or more	1868	14.70%	295	2.10%	<0.01
	country					
	Australia	321	2.50%	653	4.60%	<0.01
	Brazil	231	1.80%	423	3.00%	<0.01
	Canada	99	0.80%	398	2.80%	<0.01
	China	333	2.60%	652	4.60%	<0.01
	Denmark	77	0.60%	459	3.20%	<0.01
	Finland	53	0.40%	491	3.40%	<0.01
	France	211	1.70%	711	5.00%	<0.01
	Germany	170	1.30%	629	4.40%	<0.01
	Hong Kong	118	0.90%	271	1.90%	<0.01
	India	615	4.80%	639	4.50%	<0.01
	Indonesia	191	1.50%	460	3.20%	<0.01
	Italy	253	2.00%	650	4.50%	<0.01
	Japan	43	0.30%	251	1.80%	<0.01
	Malaysia	194	1.50%	491	3.40%	<0.01
	Mexico	127	1.00%	461	3.20%	<0.01
	Netherlands	137	1.10%	243	1.70%	<0.01
	Norway	159	1.30%	435	3.00%	<0.01
	Philippines	124	1.00%	462	3.20%	<0.01
	Saudi Arabia	1339	10.50%	398	2.80%	<0.01
	South Korea	212	1.70%	227	1.60%	<0.01
	Spain	126	1.00%	600	4.20%	<0.01
	Sweden	178	1.40%	580	4.10%	<0.01
	Taiwan	127	1.00%	449	3.10%	<0.01
	Thailand	1925	15.10%	415	2.90%	<0.01
	United Arab Emirates	1972	15.50%	376	2.60%	<0.01
	United Kingdom	68	0.50%	1013	7.10%	<0.01
	United States	488	3.80%	656	4.60%	<0.01
	Vietnam	2825	22.20%	812	5.70%	<0.01
**Lifestyle habits**						
	self-isolating	8161	64.20%	9802	68.50%	<0.01
	having difficulties isolating					
	Very easy	7586	59.70%	4589	32.10%	<0.01
	Somewhat easy	2029	16.00%	4407	30.80%	<0.01
	Neither easy nor difficult	1423	11.20%	2480	17.30%	<0.01
	Somewhat difficult	971	7.60%	1690	11.80%	<0.01
	Very difficult	361	2.80%	703	4.90%	<0.01
	Not sure	346	2.70%	436	3.00%	<0.01
	being willing to isolate					
	Very willing	7449	58.60%	8257	57.70%	<0.01
	Somewhat willing	2930	23.00%	3635	25.40%	<0.01
	Neither willing nor unwilling	1127	8.90%	1371	9.60%	<0.01
	Somewhat unwilling	641	5.00%	423	3.00%	<0.01
	Very unwilling	216	1.70%	219	1.50%	<0.01
	Not sure	353	2.80%	400	2.80%	<0.01
	whether the family had been tested	7181	56.50%	54	0.40%	<0.01
**Disease history**						
	AIDS	4736	37.20%	67	0.50%	<0.01
	arthritis	5532	43.50%	879	6.10%	<0.01
	asthma	5591	44.00%	1124	7.90%	<0.01
	cancer	4864	38.30%	389	2.70%	<0.01
	cystic fibrosis	4545	35.70%	72	0.50%	<0.01
	COPD	4902	38.50%	290	2.00%	<0.01
	diabetes	5250	41.30%	943	6.60%	<0.01
	epilepsy	4749	37.30%	113	0.80%	<0.01
	heart disease	5283	41.50%	2116	14.80%	<0.01
	hypertension	4807	37.80%	483	3.40%	<0.01
	mental disease	4837	38.00%	791	5.50%	<0.01
	multiple sclerosis	4454	35.00%	71	0.50%	<0.01
	not willing to say	471	3.70%	598	4.20%	<0.01
	no disease	3501	27.50%	8677	60.70%	<0.01
**Symptoms**						
	cough	5797	45.60%	824	5.80%	<0.01
	fever	6071	47.70%	454	3.20%	<0.01
	loss of smell	5543	43.60%	319	2.20%	<0.01
	loss of taste	5745	45.20%	321	2.20%	<0.01
	having difficulty breathing	5609	44.10%	552	3.90%	<0.01
	no symptoms	5159	40.60%	12979	90.70%	<0.01

### Descriptive statistics

This study used the Wilcoxon rank-sum test for quantitative variables such as age score and Chi-square test for proportions. This study used R language software for analysis, and all two-tailed *p* values of <0.05 were considered to be statistically significant.

### Algorithms used in this study for prediction models

To evaluate whether a given subject will be diagnosed with COVID-19 according to both geographical and lifestyle features based on the survey items, the target variable was coded 1 for cases diagnosed with COVID-19 and 0 for individuals not diagnosed with COVID-19. As the aim was a typical classification problem, this study used four types of machine learning classification models: Logistic Regression (LR), Random Forest (RF), Support Vector Machine (SVM), and Artificial Neural Network (ANN). Four machine learning models were chosen to evaluate the performance of each model and compare differences in features selected by these four models. This study randomly divided the data into an 80% training set and a 20% validation set before deploying them. Models were trained on the training dataset and verified using the validation dataset. The generalizability of the model is calculated based on the validation dataset. Four models used in this study were described below.

LR is used to classify binary categories by predicting the probabilities of outcomes. It is the most popular and simplest method applied to classification problems [18, 19]. One of the advantages of using an LR is that it is easy to understand how it operates, and it can also be applied to select important variables.

RF is an ensemble learning method for classification, and it is often viewed as the expansion of a decision tree. RF is iterated by constructing a multitude of decision trees and determining the class based on the mode of the predicted classes. That is, during training, the weight of each tree is the same. Each tree is treated as a voter, classifying one data point into one category. The majority of all trees’ decisions is the final classification of the data. The advantage of the RF is that it can avoid overfitting compared to the decision trees [20].

SVM tries to find an optimal hyperplane on which to classify data [21]. The optimal hyperplane is the perfect decision boundary for maximizing the margin between two classifications. Data on the margin line are called the support vector. The advantage of the SVM is that it can be applied to high-dimension datasets by adjusting the kernel function, but it requires more time for calculating than other models [22].

The development of an ANN is based on simulating how the human brain operates [23]. An ANN is made up of neurons with layers–one input layer, one or two hidden layers, and one output layer. Neurons in a layer connect to ones in a neighboring layer by different weights. Adjusting the weights to minimize the error function is a process used to train the model. Although training a neural network is complicated, it provides good performance of classification tasks [24].

We used the “caret package” (i.e., Classification And REgression Training), it contains functions to streamline the model training process [25]. For LR model, we used the method glm(), which has no tuning parameters; for RF model, we used the method rf(), which has the tuning parameters as mtry (#randomly selected predictors); for SVM model, we used the method svmLinear, which has the tuning parameters as c (Cost); as for ANN model, we used the default method mlp(), which has the tuning parameters as size (#Hidden Units). In this study, the ANN model was performed with 2 hidden layers. The rectified linear (relu) and softmax functions were used as the activation functions of the hidden layers and the output layers, respectively.

### Comparison of results obtained by different algorithms

Six performance matrices were used to evaluate the efficiency of the model, including the accuracy, sensitivity, specificity, positive predictive value (PPV), negative predictive value (NPV) and area under the receiver operating characteristics curve (AUROC). Accuracy is the sum of true positive and true negative predictions divided by the number of positive and negative samples. Sensitivity measures the proportion of positives that are correctly identified (i.e., the proportion of those who were correctly identified as having the condition among those who are affected). Specificity measures the proportion of negatives that are correctly identified (i.e., the proportion of those who are correctly identified as not having the condition to those who are unaffected). The PPV and NPV describe the performance of a diagnostic test or other statistical measure. A higher result can be interpreted as an indication of greater accuracy. The PPV and NPV cannot be intrinsic to the test (as true positive rates and true negative rates are); they also depend on the prevalence. The AUROC stands for the area under the receiver operating characteristic curve (ROC). That is, the AUROC measures the entire two-dimensional area underneath the entire ROC, where the ROC is a probability curve depicting the association between the true positive rate and false positive rate. By analogy, the higher the AUROC, the better the model is at distinguishing between patients with the disease and those with no disease.

### Determinants of coronavirus disease 2019 infection

To get the important variables, we used the function varImp(object = [model_name]) [26]. Basically, the default behavior is to compute the area under the ROC curve in the SVM classification models. This area is used as the measure of variable importance. For the ANN models, the basic method is used combinations of the absolute values of the weights, which was introduced by Gevrey et al. (2003) [27].

First, this study used the analytical results of the four models to identify the 15 most important features of COVID-19 infection. This study set 15 points for the first important feature of each model, 14 points for the second important feature, and so on. Then, this study calculated the total score of each important feature through a composite weighted scoring method, and finally sorted the total scores from high to low.

## Results

Table 1 shows differences between the two groups in various continuous variables. Compared to non-infected patients, infected patients were younger. This study found that compared to non-infected patients, infected patients had a lower number of times washing, number of times washing with sanitizer, frequency of cleaning, frequency of mask wearing, and number of times contacting people outside the home, and lower rates of eating alone, sleeping alone, avoiding having guests, avoiding going outside, avoiding going to shops, avoiding going to the hospital, avoiding taking public transportation, avoiding small social gatherings, avoiding medium-sized social gatherings, and avoiding touching objects.

Table 2 shows differences between the two groups in various categorical variables. Compared to non-infected patients, infected patients had a higher proportion of males, number of people (or children) in the house, a history of various diseases, and all symptoms. Countries with the highest proportions of infected patients and more than 10% of all cases included Vietnam, the United Arab Emirates, Thailand, and Saudi Arabia.

**Fig 1:**
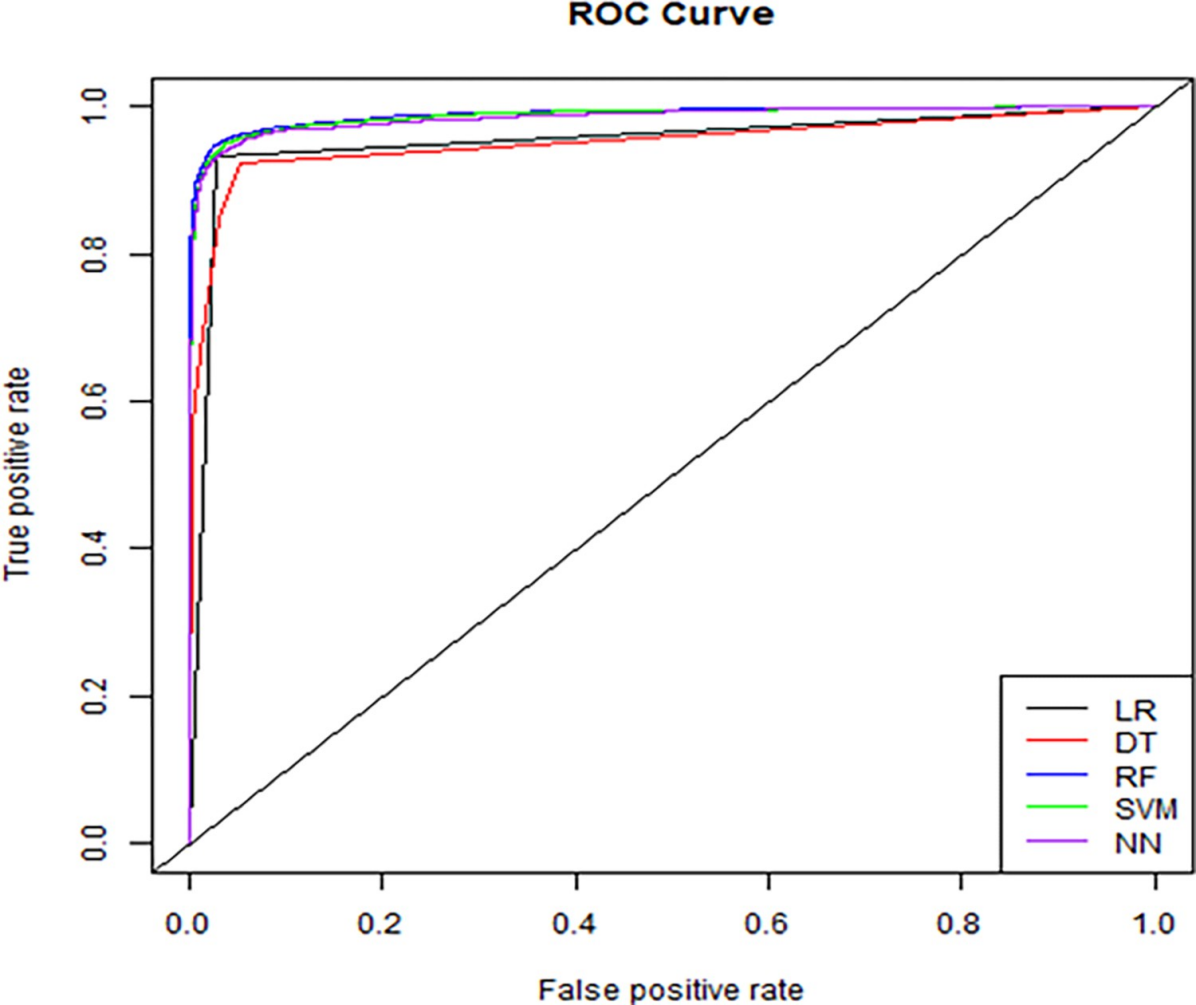
ROC curve. LR = logistic regression; DT = decision tree; RF = random forest; SVM = support vector machine; NN = artificial neural network.

Table 3 shows the accuracy, sensitivity, specificity, PPV, NPV, and AUROC of the four prediction models. It was found that the accuracy of the RF model was the highest (0.957); the SVM had the highest sensitivity (0.967); the LR had the highest specificity (0.968); the LR had the highest PPV (0.963); the SVM had the highest NPV (0.972). The RF had the highest AUROC (0.988), followed by the SVM (0.987), ANN (0.986), and LR (0.953). The ROC curve in Fig 1 shows that values of the AUROC of the RF, SVM, and ANN were the best and were similar. Although the AUROC of the LR was lower than those of the other models, its AUROC was still >95%.

**Table 3:** Machine learning model indices.

Index	LR	RF	SVM	ANN
Accuracy	0.952	0.957*	0.953	0.953
Sensitivity	0.935	0.957	0.967*	0.963
Specificity	0.968*	0.959	0.94	0.942
PPV	0.963*	0.954	0.931	0.934
NPV	0.943	0.96	0.972*	0.968
AUROC	0.953	0.988*	0.987	0.986

LR = logistic regression; RF = random forest; SVM = support vector machine; ANN = artificial neural network.

*: the best performing model for each index

Table 4 summarizes the 15 most important variables of COVID-19 infection based on the four algorithms. “Whether the family had been tested” is the top 1 variable in all models, and “no symptoms” ranks the second variable for LR, RF and ANN models. After weighting, “Whether the family had been tested” is the most critical factor, which suggests that at least one family member who had been exposed and tested for COVID-19 and this was a strong predictor for COVID-19 infection among respondents. This was followed by “no symptoms”, “loss of smell”, “loss of taste”, “epilepsy”, “AIDS”, “cystic fibrosis”, “sleeping alone”, “country” and “the number of times of leaving home in a day” (see Table 5).

**Table 4:** Variables by importance in four models.

	LR	RF	SVM	ANN
1	[2] whether the family had been tested	[2] whether the family had been tested	[2] whether the family had been tested	[3] whether the family had been tested
2	[4] no symptoms*	[4] no symptoms	[2] number of times of leaving home in a day	[4] no symptoms*
3	[2] sleeping alone	[1] country	[2] number of times of washing	[3] multiple sclerosis
4	[1] Thailand	[2] sleeping alone	[2] frequency of covering the nose and mouth	[3] cystic fibrosis
5	[3] epilepsy	[4] loss of taste	[3] COPD	[4] loss of smell
6	[2] number of times of leaving home in a day	[4] fever	[2] avoiding crowded areas	[3] epilepsy
7	[3] cystic fibrosis	[4] loss of smell	[3] AIDS	[3] AIDS
8	[4] loss of smell	[2] eat alone	[4] loss of smell	[4] loss of taste
9	[1] age*	[3] AIDS	[2] soap washing	[3] cancer
10	[4] loss of taste	[3] epilepsy	[4] loss of taste	[3] heart disease
11	[3] cancer	[4] having difficulty breathing	[1] the number of contacts with people inside the home	[2] frequency of cleaning
12	[3] arthritis	[4] cough	[3] cancer	[3] COPD
13	[3] AIDS	[3] cystic fibrosis	[3] cystic fibrosis	[3] arthritis
14	[3] multiple sclerosis	[3] COPD	[3] epilepsy	[4] fever
15	[3] COPD	[2] number of times of leaving home in a day	[2] avoiding medium-sized social gatherings	[2] frequency of covering the nose and mouth

LR = logistic regression; RF = random forest; SVM = support vector machine; ANN = artificial neural network.

Four types: [1] Basic characteristics [2] Lifestyle habits [3] Disease history [4] Symptom

*: means negative correlation

**Table 5:** Weighted importance of variables by model.

Categories	Variables	LR	RF	SVM	ANN	Total
Lifestyle habits	whether the family had been tested	15	15	15	15	60
Symptoms	no symptoms	14	14	0	14	42
Symptoms	loss of smell	8	9	8	11	36
Symptoms	loss of taste	6	11	6	8	31
Disease history	epilepsy	11	6	2	10	29
Disease history	AIDS	3	7	9	9	28
Disease history	cystic fibrosis	9	3	3	12	27
Lifestyle habits	sleeping alone	13	12	0	0	25
Basic characteristics	country	12	13	0	0	25
Lifestyle habits	the number of times of leaving home in a day	10	1	14	0	25
Disease history	COPD	1	2	11	4	18
Disease history	cancer	5	0	4	7	16
Disease history	multiple sclerosis	2	0	0	13	15
Lifestyle habits	number of times of washing	0	0	13	0	13
Lifestyle habits	frequency of covering the nose and mouth	0	0	12	1	13
Symptoms	fever	0	10	0	2	12
Lifestyle habits	avoiding crowded areas	0	0	10	0	10
Disease history	arthritis	4	0	0	3	7
Basic characteristics	age	7	0	0	0	7
Lifestyle habits	soap washing	0	0	7	0	7
Disease history	heart disease	0	0	0	6	6
Lifestyle habits	eating alone	0	5	0	0	5
Symptoms	having difficulty breathing	0	5	0	0	5
Lifestyle habits	frequency of cleaning	0	0	0	5	5
Symptoms	cough	0	4	0	0	4
Lifestyle habits	avoiding medium-sized social gatherings	0	0	1	0	1
Basic characteristics	the number of contacts with people outside the home	0	0	0	0	0

LR = logistic regression; RF = random forest; SVM = support vector machine; ANN = artificial neural network.

## Discussion

This is one of the first studies to use huge amounts of survey data from 28 countries (with 315,276 interviewees) that involved basic characteristics, lifestyle, disease history, and COVID-19 symptoms and AI technologies to predict COVID-19 infection. The AUROC of each model is between 0.951–0.988, and the RF model has the highest AUROC (0.988). The prediction accuracy of all modules are higher than 93%, with high sensitivity (≧91%) and high specificity (≧94%). Among them, the RF’s accuracy rate (95.7%) was the highest. The results pointed out that the most important factors of COVID-19 infection were, in order, whether the family had been tested, having no symptoms, loss of smell, loss of taste, a history of epilepsy, AIDS and cystic fibrosis.

Compared to high-cost and difficult-to-access medical imaging data, this study used a questionnaire survey based on basic characteristics and behaviors of individuals across many countries, and used AI machine learning methods to obtain very high accuracy rates (93%~96%) for COVID-19 infection prediction modules. This study included four major categories of variables, including basic characteristics, lifestyle habits, disease histories, and symptoms, with a total of 52 variables. These variables provide a complete and detailed discussion of multiple factors possibly affecting COVID-19 infection.

Based on the findings, this study recommend the following for COVID-19 prevention in countries around the world. (1) Age: Young people are more susceptible to infection, possibly because they have more opportunities to socialize and contact others. (2) High-risk groups based on medical history (prevention): People with a history of epilepsy, AIDS or cystic fibrosis should pay special attention. (3) High-risk groups based on symptoms (emergency): Patients with symptoms of loss of smell and loss of taste should pay more attention. (4) The importance of screening when the person is exposed: people who have family members being tested are more likely to be found to be infected. (5) Lifestyle recommendations: individuals who sleep alone and leave home less often might reduce COVID-19 infection risk.

This study has several limitations. The data source of the study was a questionnaire survey across 28 countries. The study was based on survey responses, which is vulnerable to recall bias and underestimation attributable to bias of detection and reporting of COVID-19 infection. Further, this study is a secondary analysis of existing data sourced from an international survey. Therefore, the analysis and findings are restricted to the range of information and level of details collected by the original survey. The survey may underrepresent the most socially disadvantaged individuals and those in remote areas, particularly those without phones, speaking other languages or whose health limited their participation. Possible sources of non-sampling error of the original survey might include non-response bias, and cultural differences in question interpretation. While the analysis provides insights into behaviors for preventing COVID-19 infection, this study did not assess the actual effects of the recommended behaviors to avoid infection (such as leaving the home less often), which is beyond the scope of this study. Moreover, this study did not have information on the severity or the outcome of COVID-19 infection (such as death). Future studies are warranted to predict severe COVID-19 infection and predict COVID-related mortality. Finally, this study did not have information for developing prediction models specific to regions and ethnic groups [28]; this should be an important area for future research as it may be informative for prevention strategy development. Nevertheless, the AI models with big data can be an exemplar for disease risk prediction.

## Conclusions

To date, the health, life, and economy of people in all countries around the world are still being greatly affected by the COVID-19 pandemic. This study used an international survey data including disease history and lifestyle habits and AI methods to predict COVID-19 infection. The findings provide insights that young people, those with a history of epilepsy, AIDS or cystic fibrosis, and those with symptoms such as loss of smell, loss of taste, etc., have high-risk for COVID-19 infection. Important prevention behaviors include COVID screening (especially when a family member is being tested for COVID), sleeping alone, and leaving home less often. These findings can be applied to real applications, including ways to help identify high-risk groups and ways to avoid COVID-19 infection through changes in lifestyle habits.

## Supporting information

S1 Appendix Variables type and description. (DOC)
