# Supplementary material for: Determinants of coronavirus disease 2019 infection by artificial intelligence technology: A study of 28 countries
Source: PLoS One. 2022 Aug 26;17(8):e0272546. doi: 10.1371/journal.pone.0272546 (PMC9417026; doi:10.1371/journal.pone.0272546)
Supplement: S1 Appendix — (DOC) [file pone.0272546.s001.doc]

**Appendix.** **Variables type and description**

| **Category** | **Variable** | **Description** | **Index** | **Type** |
| --- | --- | --- | --- | --- |
| Basic characteristics | gender |  | male/female | Factor |
|  | age |  |  | Numeric |
|  | weight |  |  | Numeric |
|  | number of people in the household | number of all people in household |  | Factor |
|  | number of children in the household | number of children under 18 in household | 0 or not sure, 1, 2, 3, 4, 5, 6 or more | Factor |
|  | country | Australia, Brazil, Canada, China, Denmark, Finland, France, Germany, Hong Kong, India, Indonesia, Italy, Japan, Malaysia, Mexico, Netherlands, Norway, Philippines, Saudi Arabia, South Korea, Spain, Sweden, Taiwan, Thailand, United Arab Emirates, United Kingdom, United States | Yes/No | Factor |
| Lifestyle habits | number of times of washing | number of times you washed your hands with soap or used hand sanitizer |  | Numeric |
|  | sanitizer washing | How often you used hand sanitizer? | always, frequently, sometimes, rarely, not at all | Numeric |
|  | soap washing | How often you washed hands with soap and water? | always, frequently, sometimes, rarely, not at all | Numeric |
|  | frequency of cleaning | cleaned frequently touched surfaces in the home | always, frequently, sometimes, rarely, not at all | Numeric |
|  | eating alone | eat separately at home, when normally you would eat a meal with others | always, frequently, sometimes, rarely, not at all | Numeric |
|  | sleeping alone | slept in separate bedrooms at home, when normally you would share a bedroom | always, frequently, sometimes, rarely, not at all | Numeric |
|  | frequency of mask wearing | How often you worn a face mask outside your home? | always, frequently, sometimes, rarely, not at all | Numeric |
|  | frequency of covering the nose and mouth | How often you covered your nose and mouth when sneezing or coughing? | always, frequently, sometimes, rarely, not at all | Numeric |
|  | the number of contacts with people inside the home | number of people you contact with inside the house (within 2 meters / 6 feet) |  | Numeric |
|  | the number of contacts with people outside the home | number of people you contact with outside the house (within 2 meters / 6 feet) |  | Numeric |
|  | number of times of leaving home in a day | number of times you leave your home/where you are staying yesterday |  | Numeric |
|  | avoiding having guests | avoided having guests to your home | always, frequently, sometimes, rarely, not at all | Numeric |
|  | avoiding contacting people | avoided contact with people who have symptoms or you think may have been exposed to the coronavirus | always, frequently, sometimes, rarely, not at all | Numeric |
|  | avoiding going outside | avoided going out in general | always, frequently, sometimes, rarely, not at all | Numeric |
|  | avoiding going to shops | avoided going to shops | always, frequently, sometimes, rarely, not at all | Numeric |
|  | avoiding going to the hospital | avoided going to hospital or other healthcare settings | always, frequently, sometimes, rarely, not at all | Numeric |
|  | avoiding taking public transportation | avoided taking public transport | always, frequently, sometimes, rarely, not at all | Numeric |
|  | avoiding small social gatherings | avoided small social gatherings (not more than 2 people) | always, frequently, sometimes, rarely, not at all | Numeric |
|  | avoiding medium-sized social gatherings | avoided medium-sized social gatherings (between 3 and 10 people) | always, frequently, sometimes, rarely, not at all | Numeric |
|  | avoiding large-sized social gatherings | avoided large-sized social gatherings (more than 10 people) | always, frequently, sometimes, rarely, not at all | Numeric |
|  | avoiding crowded areas | avoided crowded areas | always, frequently, sometimes, rarely, not at all | Numeric |
|  | avoiding touching objects | avoided touching objects in public | A always, frequently, sometimes, rarely, not at all | Numeric |
|  | self-isolating | In the last 7 days, would you isolate yourself after feeling unwell or having any of the above new symptoms? | Yes/No | Factor |
|  | having difficulties of isolating | the extent of difficulty for you to self-isolate for 7 days | very easy, somewhat easy, neither easy nor difficult, somewhat difficult, very difficult, not sure | Factor |
|  | being willing to isolate | the extent you are willing or not to self-isolate for 7 days | very willing, somewhat willing, neither willing nor unwilling, neither willing nor unwilling, somewhat unwilling, very unwilling, not sure | Factor |
|  | whether the family had been tested | in the last 7 days, has someone in your household been tested for coronavirus (COVID-19)? | Yes and they tested positive, others | Factor |
| Disease history | AIDS  arthritis  asthma  cancer  cystic fibrosis COPD  diabetes  epilepsy  heart disease  hyperlipidemia  hypertension  mental disease  multiple sclerosis  no disease  no willing to say | Have you been diagnosed with these diseases? | Yes/No | Factor |
| Symptoms | cough  fever  loss of smell  loss of taste  having difficulty breathing  no symptoms | In the last 7 days, have you experienced these symptoms? | Yes/No | Factor |

AIDS = Acquired Immunodeficiency Syndrome; COPD = Chronic Obstructive Pulmonary Disease
